# Role of Outer Membrane Vesicles From *Helicobacter pylori* in Atherosclerosis

**DOI:** 10.3389/fcell.2021.673993

**Published:** 2021-11-01

**Authors:** Na Wang, Faying Zhou, Caiyu Chen, Hao Luo, Jingwen Guo, Wei Wang, Jian Yang, Liangpeng Li

**Affiliations:** ^1^Department of Cardiology, Daping Hospital, The Third Military Medical University, Chongqing, China; ^2^Chongqing Key Laboratory of Hypertension Research, Chongqing Institute of Cardiology, Chongqing, China; ^3^Department of Neurology and Centre for Clinical Neuroscience, Daping Hospital, The Third Military Medical University, Chongqing, China; ^4^Department of Clinical Nutrition, The Third Affiliated Hospital of Chongqing Medical University, Chongqing, China

**Keywords:** *H. pylori*, outer membrane vesicles, atherosclerosis, endothelial cells, CagA, lipopolysaccharide, inflammatory factor

## Abstract

Infection is thought to be involved in the pathogenesis of atherosclerosis. Studies have shown the association between *helicobacter pylori* (*H. pylori*) and coronary artery disease. It is interesting to find *H. pylori* DNA and cytotoxin-associated gene A (CagA) protein in atherosclerotic plaque. Outer membrane vesicles (OMVs), secreted by *H. pylori*, exert effects in the distant organ or tissue. However, whether or not OMVs from *H. pylori* are involved in the pathogenesis of atherosclerosis remains unknown. Our present study found that treatment with OMVs from CagA-positive *H. pylori* accelerated atherosclerosis plaque formation in ApoE^–/–^ mice. *H. pylori*-derived OMVs inhibited proliferation and promoted apoptosis of human umbilical vein endothelial cells (HUVECs), which was also reflected in *in vivo* studies. These effects were normalized to some degree after treatment with lipopolysaccharide (LPS)-depleted CagA-positive OMVs or CagA-negative OMVs. Treatment with *H. pylori*-derived OMVs increased reactive oxygen species (ROS) levels and enhanced the activation of nuclear factor-κB (NF-κB) in HUVECs, which were reversed to some degree in the presence of a superoxide dismutase mimetic TEMPOL and a NF-κB inhibitor BAY11-7082. Expressions of interleukin-6 (IL-6) and tumor necrosis factor alpha (TNF-α), two inflammatory factors, were augmented after treatment with OMVs from *H. pylori.* These suggest that *H. pylori*-derived OMVs accelerate atherosclerosis plaque formation via endothelium injury. CagA and LPS from *H. pylori*-OMVs, at least in part, participate in these processes, which may be involved with the activation of ROS/NF-κB signaling pathway. These may provide a novel strategy to reduce the incidence and development of atherosclerosis.

## Introduction

Cardiovascular disease is a major cause of death worldwide (Myers and Mendis, 2014). Atherosclerosis, a chronic metabolic or inflammatory disease, is characterized by accumulation of cholesterol and recruitment of macrophages in the arterial wall. The intact endothelial layer is important to keep the proliferation, migration and apoptosis of vascular smooth muscle cells (VSMCs) in the normal range. Therefore, endothelial injury is thought to be involved in the development of atherosclerosis (Ali et al., 2013).

Nowadays, the relationship between infection and coronary heart disease (CAD) has attracted more and more attention. Infection of bacteria from the oral cavity, and perhaps even the gut, correlates with atherosclerosis (Koren et al., 2011). *Helicobacter pylori* (*H. pylori*) infection is the most common infection in the world, particularly in the developing countries. Previous studies have shown the positive relationship between *H. pylori* and CAD (Rožanković et al., 2011). However, how *H. pylori* are involved in the pathogenesis of CAD is still unknown. It is interesting to find the *H. pylori* DNA and cytotoxin-associated gene A (CagA) protein in atherosclerotic plaque (Kowalski, 2001). As one kind of bacteria in the stomach, how *H. pylori* escapes from the defensive system in blood and goes to atherosclerotic plaque remains unknown.

*H. pylori*, gram-negative bacteria, exhibits tropism toward the gastric epithelium (Cover and Peek, 2013). The mechanisms underlying *H. pylori* infection-induced tissue injury are not clearly established. Several studies showed that *H. pyloric* release outer membrane vesicles (OMVs) *in vitro* and *in vivo* (Chmiela et al., 2018; Jarzab et al., 2020). These OMVs, contained proteins, lipopolysaccharide (LPS), and lipoproteins, could be transferred to the cytosol and nucleus in the recipient cells even at a distance (Keenan et al., 2008; Parker et al., 2010). A growing body of evidence indicates that OMVs have important physiological functions, including signal transduction, immune regulation, cell adhesion, blood coagulation, and induction of inflammation (Hodges and Hecht, 2012; Davies et al., 2019). However, whether or not OMVs from *H. pylori* are involved in the pathogenesis of atherosclerosis is still unknown. In the present study, we test the hypothesis that OMVs from *H. pylori* accelerate atherosclerosis plaque formation via endothelium injury. Furthermore, we found that CagA and LPS from *H. pylori*-derived OMVs, at least in part, participate in OMVs-induced proliferation inhibition and apoptosis in human umbilical vein endothelial cells (HUVECs). These may provide a novel strategy to reduce the incidence and development of atherosclerosis.

## Materials and Methods

### Bacterial and Cell Culture

The well-characterized *H. pylori* clinical isolates were used in this study. *H. pylori* 26695 (CagA-positive) and *H. pylori* NCTC11637 (CagA-negative), kindly offered by Prof. Qinghua Xie (Department of Microbiology and Biochemical Pharmacy, College of Pharmacy and Laboratory Medicine, The Third Military Medical University), were grown on blood agar plates (10% rabbit blood, 5% glucose, 10 μg/mL norvancomycin hydrochloride, 2 μg/mL amphotericin B, 5 μg/mL trimethoprim, 0.38 μg/mL polymyxin B). For liquid cultures, bacterial growth from one plate was harvested and inoculated into a 100 mL volume of Skirrow’s medium supplemented with 10% newborn calf serum, 5% glucose, 10 μg/mL norvancomycin hydrochloride, 2 μg/mL amphotericin B, 5 μg/mL trimethoprim, 0.38 μg/mL polymyxin B. Cultures were incubated at 37°C in a microaerobic environment (5% O_2_, 10% CO_2_, and 85% N_2_) with constant rotation (120 rpm) (Yamaoka et al., 1997).

HUVECs, purchased from American Type Culture Collection, were cultured with Dulbecco’s modified eagle medium with 10% fetal bovine serum, penicillin (100 IU/mL), and streptomycin (100 μg/mL) at 37°C in a humidified atmosphere of 5% CO_2_ and 95% air. HUVECs were incubated with control (vehicle), OMVs (10 μg/mL) with or without a superoxide dismutase mimetic TEMPOL (4-hydroxy-2,2,6,6-tetramethylpiperidinyl-1-oxyl) or a nuclear factor-κB (NF-κB) inhibitor BAY11-7082 for 24 h.

### Harvesting and Identifying of Outer Membrane Vesicles From *H. pylori*

After 72 h incubation, bacteria were removed from the broth cultures by two centrifugations (10,000 g, 15 min, 4°C). The culture supernatants were then ultracentrifuged (100,000 g, 2 h, 4°C) to recover OMVs. Resultant OMV pellets were washed twice in sterile phosphate-buffered saline (PBS), and followed with being assayed for protein content using a modification of the Lowry procedure. Samples were stored at −20°C until required (Chitcholtan et al., 2008). Then, OMVs with indicating concentrations from *H. pylori* were co-cultured with HUVECs for 24 h. The OMV concentration was indicated by its protein concentration. The content of LPS was measured according to the instruction of a commercial test kit (Wuhao Biotechnology Co., Ltd., Shanghai, China). For LPS depletion, 200 ng/mL polymyxin B, a well-known inhibitor of activation properties of LPS (Pérez-Pérez et al., 1995), was pre-treated with OMVs for 1 h at 37°C before the co-culture of OMVs and HUVECs.

### Animals and Outer Membrane Vesicles Treatment

Seven- to eight-week-old ApoE^–/–^ male mice were provided by the Experimental Animal Center of Daping Hospital (Chongqing, China). All procedures were approved by the Daping Hospital Animal Use and Care Committee. All experiments conformed to the guidelines of the ethical use of animals, and all efforts were made to minimize animal suffering and reduce the number of animals used.

All mice were housed in cages with free access to feed and water at 25°C, 60 ± 10% humidity, and a 12 h light/dark photoperiod. The mice were assigned to control or OMVs group when they started the high-fat and high-cholesterol diet containing 21% fat and 0.15% cholesterol. ApoE^–/–^ mice were intragastrically administered with vehicle or OMVs from *H. pylori* 200 μL/day for 4 weeks. At the end of 12-week-old, the mice were anesthetized and sacrificed by cervical dislocation.

### Measurement of Atherosclerotic Lesions in the Aorta

To measure the extent of atherosclerosis in the aorta, the entire aorta was removed, and then placed in 4% paraformaldehyde. The fixed aorta was stained with Oil Red O to delineate the atherosclerotic lesions, and images were obtained with a light microscope. The Oil Red O-positive area and total aortic surface area were measured using Image J software. Atherosclerotic lesions of the aorta were defined as the percentage of Oil Red O-positive area to total aortic surface area, which was presented as fold changes compared to the control group in each set.

### Proliferation Assay

Cell proliferation was analyzed using a Cell Counting Kit (CCK-8) (Dojindo, Kuma-moto, Japan) according to the manufacturer’s instructions. Briefly, 1 × 10^4^ cells were inoculated into 96-well plates. After 60% confluence, HUVECs were induced quiescent in serum-free medium for 24 h. Then, 90 μL of medium and 10 μL of the CCK-8 solution were added to each well and the plate was maintained at 37°C for 1 h. Optical density (OD) values were determined at 450 nm with Varioskan Flash microplate reader (Thermo Scientific). Data are given as a percent of the control value. All experiments were repeated for six times.

[^3^H] thymidine (1 μCi/mL) was added to the growth medium of each well 6 h before the measurements. In brief, after the medium was removed, cells were treated with 0.25 mL 0.05% trypsin-0.53 mM EDTA for 5 min and diluted to 10 mL with a balanced electrolyte solution. Cells were then treated with 10% trichloroacetic acid (TCA) to precipitate acid-insoluble materials from which DNA was extracted with 0.1 mol/L NaOH. DNA was collected on a Whatman GF/B filter and washed twice with 5 mL ice-cold PBS. The filter was then cut and shaken in 3.5 mL scintillation fluid for 24 h before counting in a liquid scintillation counter (Beckman LS6500, Beckman). [^3^H]-thymidine incorporation was determined using a liquid scintillation counter.

### Determination of Apoptotic Cells

HUVECs grown on slides were fixed for 15 min in 4% paraformaldehyde. Samples were exposed to the enzymatic reaction mixture containing terminal deoxynucleotidyl transferase and fluorescein-dUTP for 2 h at 37°C and 4′,6-diamidino-2-phenylindole (DAPI) staining for about 10 s. After washing with PBS, the percentage of the terminal deoxynucleotidyl transferase-mediated dUTP nick end labeling (TUNEL)-positive cells relative to total cells was calculated by counting all cells in five random microscopic fields at a magnification of 20×.

### Immunoblotting

Cells were washed with PBS, followed by the addition of 100 μL of cell lysis buffer. Supernatants were harvested by centrifugation (15,000 g, 20 min, 4°C), and the concentrations were determined using the bicinchoninic acid method. The cytoplasmic and nuclear protein were harvested with the protein extraction kit (Beyotime, Jiangsu, China) and then subjected to immunoblotting.

Equal amount of proteins (50 μg) were subjected to SDS-PAGE with 10% polyacrylamide gel, which was followed by electrotransfer into polyvinylidene fluoride membranes and blocked with Tris-buffered saline (TBS), containing 5% non-fat dry milk for 1 h. The membranes were incubated overnight with the primary antibodies against NF-κB p65 (1:300; BD Transduction Laboratory, Minneapolis, MN, United States), IkBa (1:500; Santa Cruz Biotechnology, Santa Cruz, CA), caspase-3 (1:500; Santa Cruz Biotechnology, Santa Cruz, CA), cleaved caspase- 3 (1:500; Santa Cruz Biotechnology, Santa Cruz, CA), GAPDH (1:500, Santa Cruz Biotechnology), gp91^phox^ (1:300; BD Transduction Laboratory, Minneapolis, MN, United States), superoxide dismutase 2 (SOD2, 1:300; Santa Cruz Biotechnology, Santa Cruz, CA), and CagA (1:300; Santa Cruz Biotechnology, Santa Cruz, CA) at 4°C, and then incubated with the secondary antibodies at room temperature for 1 h. The Odyssey Infrared Imaging System (Li-COR Biosciences) was used to visualize the bands, and Quantity One image analysis software was used to analyze the relative intensities. GAPDH or caspase-3 was used to normalize the densitometric intensity for proteins.

### Dihydroethidium Staining

After treatment with *H. pylori*-derived-OMVs, HUVECs were loaded with 1 μM dihydroethidium (DHE) (Abcam, United States) and maintained for 15 min at 37°C. The intracellular levels of ROS were determined using a fluorescent microscope. The fluorescence intensity values from five different fields were quantified using ImageJ software.

### ELISA Assay

One milliliter blood were drawn from ApoE^–/–^ mice and centrifuged at 4°C and 3,000 r/min for 15 min. The supernatant was used to test inflammatory factors, including tumor necrosis factor alpha (TNF-α) and interleukin-6 (IL-6), according to the instruction of an enzyme-linked immunosorbent assay (ELISA) kit (Abcam, Cambridge, MA, United States).

### Hematoxylin-Eosin Staining

Artery segments were immersed in ice-cold 4% paraformaldehyde for 30 min. Samples were transferred to 30% sucrose–PBS solution at 4°C for 24 h, embedded in optimum cutting temperature (OCT) compound. For the microscopic quantification of the lesion area, the aorta was processed through routine steps of desiccation followed by clearing, dipping and embedding in wax, and serial sectioning (5 μm). Sections were stained with HE using the standard protocol. Then, light microscopy was used to observe the tissues.

### Statistical Analysis

The data are presented as mean ± standard deviation (SD). Comparisons within the groups were performed using ANOVA for repeated measures (or paired *t*-test when only 2 groups were compared), and comparison among groups (or *t*-test when only two groups were compared) was performed using factorial ANOVA with Holm-Sidak as the *ad hoc* test. A value of *P* < 0.05 was considered significant.

## Results

### Isolation and Identification of Outer Membrane Vesicles From *H. pylori*

**FIGURE 1:**
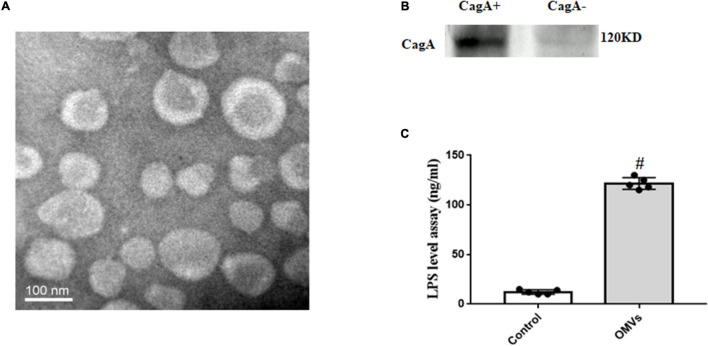
Identification and characterization of OMVs from *H. pylori*. **(A)** Electron micrographs of OMVs from *H. pylori*. Isolated OMVs from *H. pylori* were fixed with negative staining with Na-phosphotungstate, and observed by transmission electron microscope. **(B)** CagA expression in *H. pylori*-derived OMVs (CagA^+^: OMVs from CagA-positive *H. pylori*; CagA^–^: OMVs from CagA-negative *H. pylori*). **(C)** LPS levels in *H. pylori*-derived OMVs and vehicle were determined by ELISA (*n* = 5, ^#^*P* < 0.01 vs. control).

OMVs from *H. pylori* culture supernatants were isolated through a series of ultracentrifugation steps as previously described. To further identify the OMVs from *H. pylori*, electron microscopic, immunoblotting and ELISA analyses were used. As shown in Figure 1A, the electron micrograph showed that OMVs from *H. pylori* were round, with size about 20–300 nm. Immunoblotting showed the high CagA levels in OMVs from CagA-positive *H. pylori* (Figure 1B). Results of ELISA measurement also showed the substantially higher levels of LPS in *H. pylori*-derived OMVs, compared to vehicle (Figure 1C). These results identified that OMVs from *H. pylori* were correct. It should be noted that the OMVs used in our present study referred to the OMVs from CagA-positive *H. pylori.*

### *H. pylori*-Derived Outer Membrane Vesicles Accelerate Atherosclerosis Plaque Formation

**FIGURE 2:**
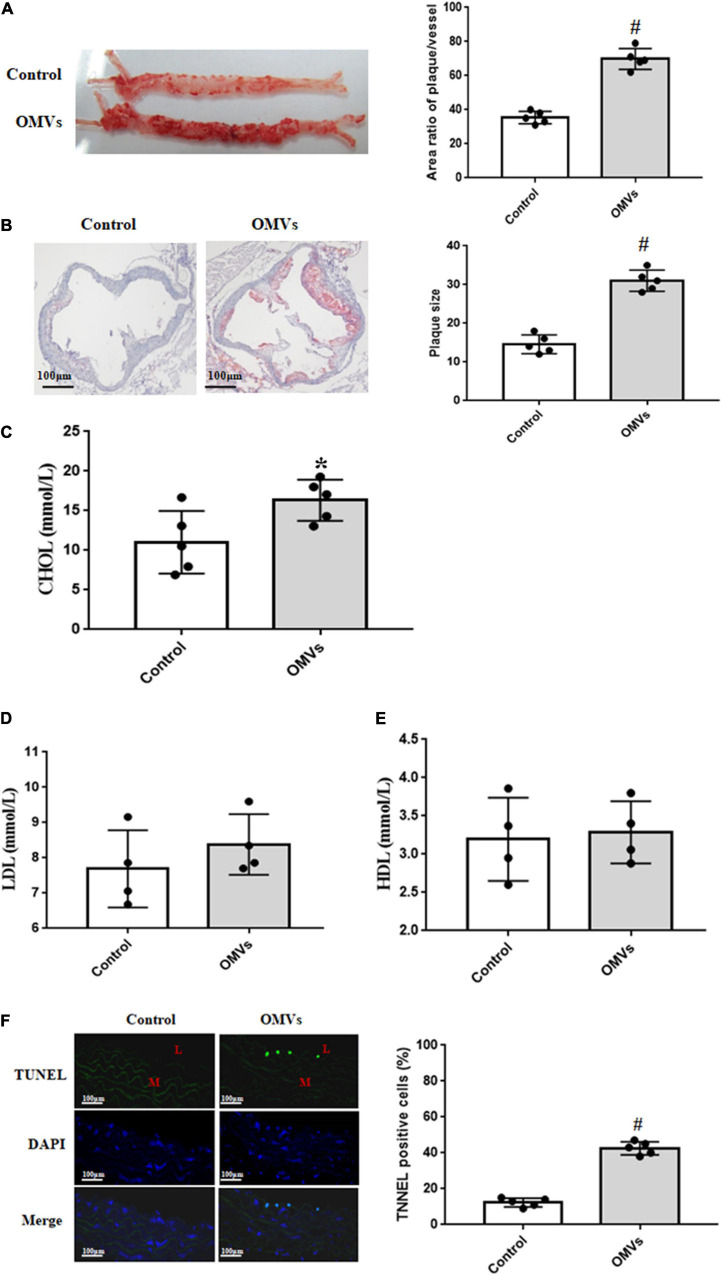
*H. pylori*-derived OMVs accelerate atherosclerosis plaque formation in ApoE^–/–^ mice. **(A)** Continuous atherosclerosis plaques were measured by Oil Red O staining in ApoE^–/–^ mice treated with *H. pylori*-derived OMVs (*n* = 5, ^#^*P* < 0.01 vs. control). **(B)** Cross-section histological analysis were determined by HE staining in ApoE^–/–^ mice treated with *H. pylori*-derived OMVs (*n* = 5, ^#^*P* < 0.01 vs. control). **(C–E)** The levels of serum lipids, including cholesterol (**C**, CHOL), low density lipoprotein cholesterol (**D**, LDL), high density lipoprotein cholesterol (**E**, HDL), were determined by ELISA in ApoE^–/–^ mice treated with *H. pylori*-derived OMVs (*n* = 4 or 5, *P < 0.05 vs. control). **(F)** Apoptosis were measured by TUNEL staining in arteries from ApoE^–/–^ and control mice. Magnification is × 20; blue, cell nuclei (DAPI staining); green, TUNEL staining; L, lumen; M, media (*n* = 5, ^#^*P* < 0.01 vs. control).

To determine the role of *H. pylori*-derived OMVs in the pathogenesis of atherosclerosis, we intragastrically administered OMVs from *H. pylori* into ApoE^–/–^ mice for 4 weeks. After examination, the aortas were cut longitudinally and respectively stained with Oil Red O and HE. Results with Oil Red O staining revealed that compared with controls, OMVs-treated ApoE^–/–^ mice had more atherosclerotic plaque (Figure 2A). This is similar with the results with HE staining, in which ApoE^–/–^ mice treated with *H. pylori*-derived OMVs had more serious atherosclerosis than control mice (Figure 2B). These indicate that *H. pylori*-derived OMVs accelerate atherosclerosis plaque formation. In addition, the serum lipid profiles were also determined. Our results showed that *H. pylori*-derived OMVs increased cholesterol levels in ApoE^–/–^ mice (Figure 2C), while the levels of serum low density lipoprotein (LDL) and high density lipoprotein (HDL) had no significant difference with control (Figures 2D,E).

Moreover, the TUNEL staining was used to check the apoptosis in arteries. Results showed that TUNEL-positive nuclei in the arterial lumen were evidently higher in arteries from OMVs-treated ApoE^–/–^ mice than that from control mice (Figure 2F). However, there was no difference in the arterial media. These suggested that the injured arterial lumen may, at least in part, participate in the *H. pylori*-derived OMVs-mediated atherosclerosis plaque formation.

### *H. pylori*-Derived Outer Membrane Vesicles Inhibit Proliferation and Promote Apoptosis of Human Umbilical Vein Endothelial Cells

**FIGURE 3:**
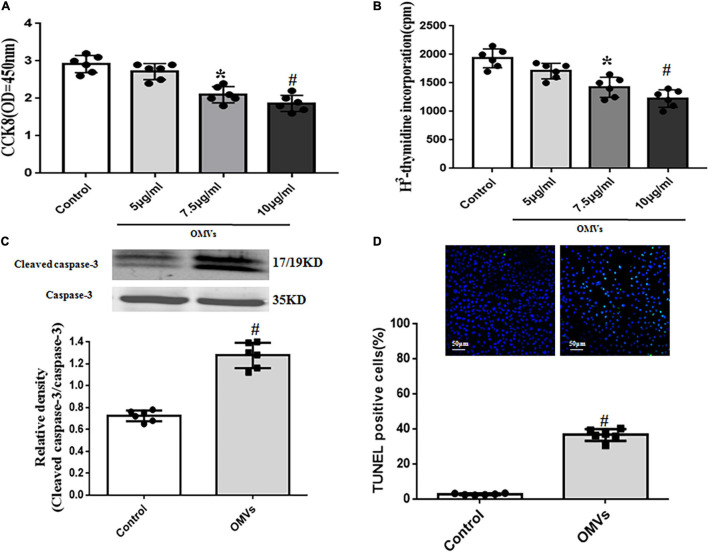
Role of *H. pylori*-derived OMVs on proliferation and apoptosis of HUVECs. **(A,B)** Proliferation of HUVECs treated with *H. pylori*-derived OMVs (5, 7.5, 10 μg/ml) were assayed by cell counting kit-8 (CCK8) **(A)** and [^3^H] thymidine incorporation **(B)**. Absorbance was detected at 450 nm (*n* = 6, *P < 0.05 or ^#^*P* < 0.01 vs. control. **(C)** Cleaved caspase-3 and total caspase-3 levels in HUVECs were determined by immunoblotting. Data were expressed as the ratio of cleaved caspase-3 to total caspase-3 expression (*n* = 6, ^#^*P* < 0.01 vs. control). **(D)** Apoptosis of HUVECs was determined by TUNEL staining. Data were expressed as the ratio of apoptotic cells to total cells (*n* = 6, ^#^*P* < 0.01 vs. control).

To investigate the possible effects of *H. pylori*-derived OMVs in the arterial lumen, the proliferation of HUVECs was examined. Via cell proliferation analysis, we found that *H. pylori*-derived OMVs suppressed the proliferation of HUVECs in a concentration-dependent manner (Figure 3A). Results from [^3^H]-thymidine incorporation also showed that *H. pylori*-derived OMVs with different concentrations exerted the inhibitory effects in HUVECs (Figure 3B). These indicated that *H. pylori*-derived OMVs inhibit HUVECs proliferation.

Next, the apoptosis of HUVECs were also determined. Results showed that incubation of HUVECs with *H. pylori*-derived OMVs (10 μg/mL) for 24 h increased the ratio of cleaved-caspase-3 to total caspase-3 expression, a common apoptosis marker (Figure 3C). We also found that compared with control, TUNEL-positive nuclei in HUVECs treated with *H. pylori*-derived OMVs were markedly increased (Figure 3D). These showed the direct evidence that *H. pylori*-derived OMVs promote apoptosis of HUVECs.

### Role of Lipopolysaccharide and Cytotoxin-Associated Gene A in the *H. pylori*-Derived Outer Membrane Vesicles -Induced Proliferation Inhibition and Apoptosis in Human Umbilical Vein Endothelial Cells

**FIGURE 4:**
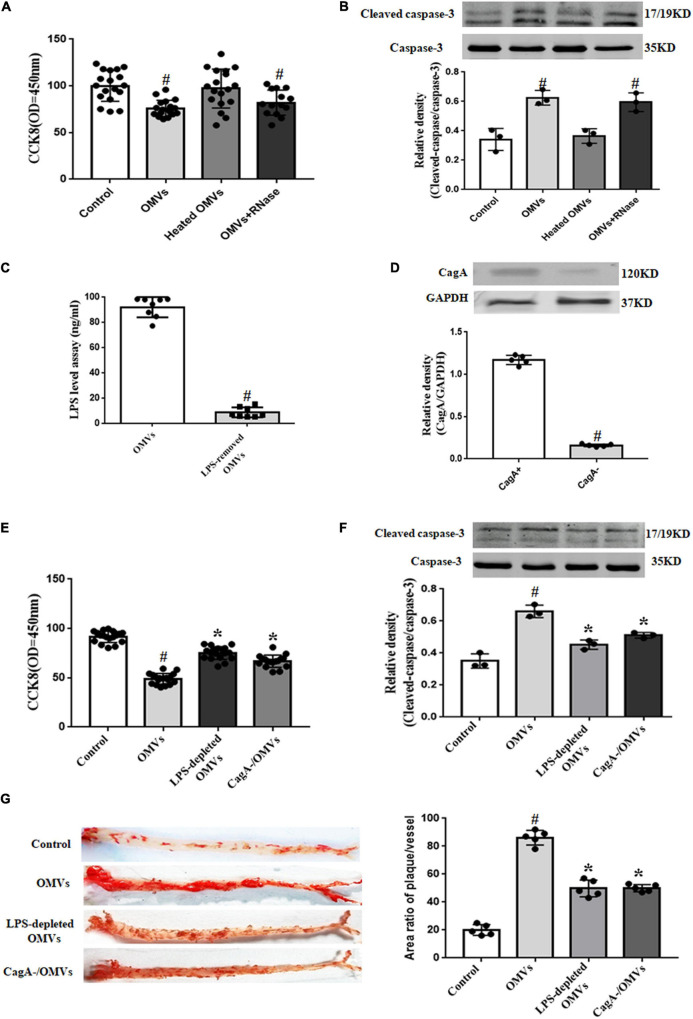
Role of LPS and CagA in *H. pylori*-derived OMVs-mediated proliferation inhibition and apoptosis in HUVECs. **(A,B)** HUVECs were administrated with control (vehicle), OMVs (10 μg/mL), heated OMVs or RNase-treated OMVs for 24 h. Proliferation of HUVECs were determined by CCK8 **(A)** (*n* = 18, ^#^*P* < 0.01 vs. control); apoptosis of HUVECs were determined by the ratio of cleaved-caspase-3 to caspase-3 expression **(B)** (*n* = 3, ^#^*P* < 0.01 vs. control). **(C)** LPS levels were determined by ELISA after treatment with polymyxin B for 1 h at 37°C (*n* = 8, ^#^*P* < 0.01 vs. OMVs). **(D)** CagA levels in CagA-positive or -negative OMVs were determined by immunoblotting (*n* = 5, ^#^*P* < 0.01 vs. CagA^+^). **(E,F)** HUVECs were administrated with control (vehicle), OMVs (10 μg/mL), LPS-depleted OMVs or CagA-negative OMVs for 24 h. Proliferation of HUVECs were determined by CCK8 **(E)** (*n* = 13, *P < 0.05 vs. OMVs or ^#^*P* < 0.01 vs. control); apoptosis of HUVECs were determined by the ratio of cleaved-caspase-3 to total caspase-3 expression **(F)** (*n* = 3, *P < 0.05 vs. OMVs or ^#^*P* < 0.01 vs. control). **(G)** Continuous atherosclerosis plaques were measured by Oil Red O staining in ApoE^–/–^ mice treated with control (vehicle), OMVs, LPS-depleted OMVs or CagA^–^ OMVs after 4 weeks (*n* = 5, *P < 0.05 vs. OMVs or ^#^*P* < 0.01 vs. control).

OMVs, varying in their abundance, size and composition, contain materials such as functional transmembrane proteins, RNAs, and DNA (Parker and Keenan, 2012). To make clear which components of OMVs affect HUVECs proliferation and apoptosis, *H. pylori*-derived OMVs were treated with Triton X-100 and then incubated with RNase to remove RNAs. Results showed that after co-treatment with RNase, the proliferation inhibition and apoptosis induced by *H. pylori*-derived-OMVs were not changed (Figures 4A,B), suggesting that proteins, other than RNAs, might participate in *H. pylori*-derived OMVs-mediated effects in HUVECs. To further confirm the role of proteins in the effects, *H. pylori*-derived OMVs were denatured after heated in 95°C for 30 min. Our results found that the proliferation inhibition and apoptosis induced by *H. pylori*-derived-OMVs were lost in HUVECs treated with the denatured OMVs (Figures 4A,B), indicating that proteins in the OMVs may participate in the effects induced by *H. pylori*-derived-OMVs in HUVECs.

It is known that the components of OMVs are complex. Among them, LPS and CagA have been reported to regulate cell proliferation and apoptosis (Valenzuela et al., 2013). To explore whether or not LPS and CagA contribute to the OMVs-mediated proliferation inhibition and apoptosis in HUVECs, LPS was depleted by treatment with polymyxin B, an inhibitor of activation properties of LPS (Figure 4C), and CagA-negative OMVs was also used (Figure 4D). Results showed that both OMVs-induced proliferation inhibition and apoptosis in HUVECs were normalized to some degree after treatment with LPS-depleted OMVs or CagA-negative OMVs (Figures 4E,F). The results in *in vitro* experiments were also reflected in *in vivo* studies. We found that compared with the ApoE^–/–^ mice treated with *H. pylori*-derived-OMVs, there were less atherosclerosis plaque formation in ApoE^–/–^ mice treated with LPS-depleted OMVs or OMVs originated from CagA-negative bacterial strain (Figure 4G). These results indicated that both LPS and CagA, at least in part, participate in the effects induced by *H. pylori*-derived-OMVs in HUVECs.

### Role of Reactive Oxygen Species/NF-κB Signaling Pathway in the *H. pylori*-Derived Outer Membrane Vesicles -Induced Proliferation Inhibition and Apoptosis in Human Umbilical Vein Endothelial Cells

**FIGURE 5:**
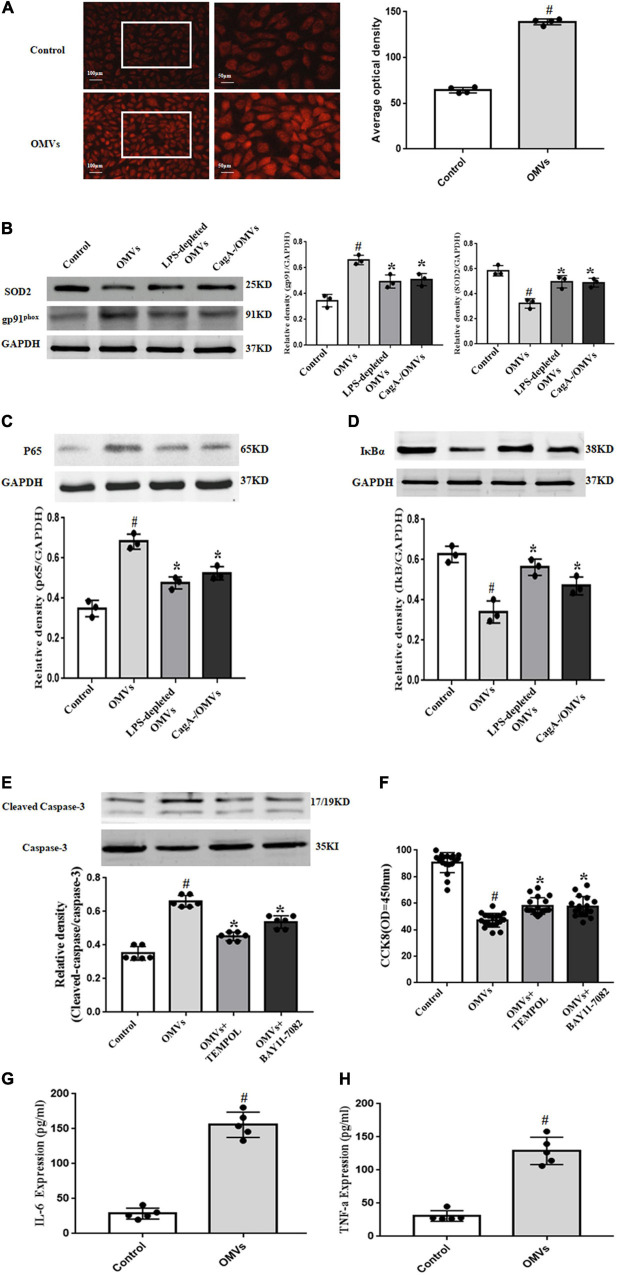
Role of ROS/NF-κB signaling pathway in *H. pylori*-derived OMVs-mediated effects in HUVECs. **(A)** ROS were determined by dihydroethidium staining in HUVECs treated with *H. pylori*-derived OMVs (10 μg/mL) for 24 h (*n* = 4, ^#^*P* < 0.01 vs. control). **(B–D)** HUVECs were administrated with control (vehicle), OMVs (10 μg/mL), LPS-depleted OMVs or CagA-negative OMVs for 24 h. The expressions of SOD2 and gp91^phox^ **(B)**, p65 **(C)**, IκBα **(D)** were determined by immunoblotting (*n* = 3, *P < 0.05 vs. OMVs or ^#^*P* < 0.01 vs. control). **(E)** HUVECs were treated with control (vehicle), OMVs (10 μg/mL), OMVs + TEMPOL, and OMVs + BAY11-7082 for 24 h. The expression of cleaved-caspase-3 and total caspase-3 were determined by immunoblotting (*n* = 6, *P < 0.05 vs. OMVs or ^#^*P* < 0.01 vs. control). **(F)** Proliferation of HUVECs were assayed by CCK8 after treatment with vehicle, OMVs (10 μg/mL), OMVs + TEMPOL, and OMVs + BAY11-7082 for 24 h (*n* = 17, *P < 0.05 vs. OMVs or ^#^*P* < 0.01 vs. control). **(G,H)** ApoE^–/–^ mice or control mice were intragastrically administered with vehicle or *H. pylori*-derived OMVs for 4 weeks. Levels of IL-6 **(G)** and TNF-α **(H)** were determined by ELISA (*n* = 5, ^#^*P* < 0.05 vs. control).

It is well known that reactive oxygen species (ROS) play a vital role in the regulation of cell proliferation and apoptosis (Brown and Griendling, 2015). In our present study, treatment with *H. pylori*-derived-OMVs (10 μg/ml, 24 h) increased ROS levels in HUVECs (Figure 5A). We also found that *H. pylori*-derived-OMVs up-regulated the expression of the NADPH oxidase protein gp91^phox^, but down-regulated the expression of the antioxidant protein SOD2, which were both normalized to some degree after treatment with polymyxin B or CagA-negative OMVs (Figure 5B). Studies have shown that increased ROS lead to the activation of NF-κB, which causes the proliferation inhibition and apoptosis of HUVECs (Jang et al., 2017; Safi et al., 2018). Thus, the NF-κB activity in HUVECs was investigated. We found that *H. pylori*-derived-OMVs increased the protein expression of NF-κB p65 subunit, but decreased the protein levels of IκBα, an inhibitor of NF-κBα, which were normalized to some degree after administration with LPS-depleted OMVs or CagA-negative OMVs (Figures 5C,D).

To further define the role of ROS and NF-κB in the effects of *H. pylori*-derived-OMVs, a superoxide dismutase mimetic TEMPOL and a NF-κB inhibitor BAY11-7082 were used. We found that *H. pylori*-derived-OMVs increased the ratio of cleaved-caspase-3 to caspase-3 expression and decreased the proliferation of HUVECs, which were normalized to some degree in the presence of TEMPOL or BAY11-7082 (Figures 5E,F). In addition, the levels of inflammatory factors, downstream targets of NF-κB, were also determined. Results showed that compared with the control, the expressions of IL-6 and TNF-α were significantly augmented after treatment with *H. pylori*-derived-OMVs (Figures 5G,H). These results indicated that activation of ROS/NF-κB signaling pathway may be involved in the proliferation inhibition and apoptosis induced by *H. pylori*-derived-OMVs in HUVECs.

## Discussion

Atherosclerosis is a complex disease process with multiple risk factors (von Scheidt et al., 2021). Up to now, the exact mechanism of processes leading to initiation of atherosclerotic lesions has remained unclear. Clinical and experimental studies have shown that specific pathogens, including chlamydia pneumoniae, mycobacterium tuberculosis, human immunodeficiency virus, may promote to the development of atherosclerosis and the occurrence of clinical events (Tumurkhuu et al., 2018; Zhao et al., 2019). Therefore, the possible proatherogenic roles of common infectious pathogens are worthy of further exploration.

*H. pylori*, one of the most common bacterial pathogens in human beings, colonizes the gastric mucosa. In recent years, many studies have demonstrated the associations between *H. pylori* infection and cardiovascular diseases such as atherosclerosis. Epidemiological studies have suggested an association between *H. pylori* infection and atherosclerosis as a common clinical feature of atherosclerosis (Matsuo et al., 2017). *H. pylori*-specific DNA had been detected in atheromatous plaque (Kowalski, 2001). Patients with *H. pylori* infection are about three times more at risk of CAD (Fang et al., 2019). However, how *H. pylori* escapes from the defensive system and goes to atherosclerotic plaque is still unknown.

Extracellular vesicles (EVs), membrane-enclosed structures released from prokaryotic and eukaryotic cells, play a vital role to promote intercellular communication (Yang et al., 2020). EVs contain different materials such as RNAs, proteins, DNA, microRNA, and lipids, which can be delivered from the cell of origin to a recipient cell, which is in the vicinity or distant from the cell of origin (Jansen et al., 2017). *H. pylori* release parts of its outer membrane in the form of vesicles. These OMVs released by several gram-negative bacteria contain numerous bacterial antigens and virulence factors (Jarzab et al., 2020). *H. pylori*-derived OMVs are small, circular structures with intact outer membranes that are shed from the surface (Fiocca et al., 1999). Studies have reported that OMVs derived from bacteria play an important role in the pathogenesis of cardiovascular diseases such as atherosclerosis and thromboembolism (Anderson et al., 2010). For example, oral bacteria *Porphyromonas gingivalis*-derived OMVs promote calcification of VSMCs and induce the inflammatory responses in HUVECs (Ho et al., 2016; Yang et al., 2016). In our present study, *H. pylori*-derived OMVs were isolated and identified by different methods. Both Oil Red O and HE staining showed that ApoE^–/–^ mice intragastrically administered with *H. pylori*-derived OMVs had more serious atherosclerosis, indicating that *H. pylori*-derived OMVs accelerate atherosclerosis plaque formation. We also found that injured arterial lumen may participate in the *H. pylori*-derived OMVs-mediated atherosclerosis plaque formation, which was verified by further *in vitro* studies, in which *H. pylori*-derived OMVs inhibited proliferation and promoted apoptosis of HUVECs.

*H. pylori*-derived OMVs contain some materials including CagA and LPS, which are associated with cardiovascular diseases such as atherosclerosis. CagA-positive *H. pylori* infection impairs endothelial function in patients and mice through exosome-mediated mechanisms (Xia et al., 2020), promotes coronary atherosclerosis via increasing serum oxidized LDL and high-sensitivity C-reactive protein in patients with CAD (Huang et al., 2011). Chronic infection by CagA-positive *H. pylori* correlates with high circulating levels of B-type natriuretic peptide and IL-6 in patients with acute CAD (Figura et al., 2014). Studies have also shown that CagA *per se* exerts functions in the progression of atherosclerosis. For example, exosomal CagA derived from *H. pylori*-infected gastric epithelial cells induces macrophage foam cell formation and promotes atherosclerosis (Yang et al., 2019). On the other hand, it is well accepted that LPS aggravates atherosclerosis via evoking a systemic inflammation (Ostos et al., 2002; Gitlin and Loftin, 2009). In our present study, OMVs-induced proliferation inhibition and apoptosis in HUVECs were reversed after treatment with LPS-depleted OMVs or CagA-negative OMVs. These were reflected in *in vivo* studies, which showed that there was less atherosclerosis plaque formation in ApoE^–/–^ mice treated with LPS-depleted OMVs or CagA-negative OMVs. These indicated that CagA and LPS participate in the proliferation inhibition and apoptosis in HUVECs induced by *H. pylori*-derived-OMVs.

ROS/NF-κB signaling pathway is involved in the development of atherosclerosis (Ellulu et al., 2016; Zhang et al., 2016). OMVs exert physiological functions via regulating ROS levels. For example, *Porphyromonas gingivalis*-derived OMVs induce a shift in macrophage metabolism from oxidative phosphorylation to glycolysis, increase mitochondrial ROS production, causing pyroptotic cell death in macrophages (Fleetwood et al., 2017). *Borrelia burgdorferi* originated-OMVs serve to directly counter superoxide production via SOD2 expression in human neuroblastoma cells (Wawrzeniak et al., 2020). OMVs from *Proteus mirabilis* regulate the function of boar sperm via increasing ROS levels and inducing sperm membrane reconstruction (Gao et al., 2018). Studies have also shown the regulation of OMVs on the activation of NF-κB. *H. pylori*-derived OMVs stimulate IL-8 secretion through NF-κB activation in two human gastric epithelial cell lines and a human monocytic cell line (Choi et al., 2020). OMVs from non-pathogenic *Escherichia (E.) coli* or pathogenic *E. coli* trigger NF-κB translocation to the nucleus, resulting in up-regulation of adhesion molecules and cytokines, and then initiating the inflammatory cascade in endothelial cells (Soult et al., 2013). In our present study, we found that *H. pylori*-derived OMVs increased ROS levels and activated NF-κB in HUVECs, which were, respectively, normalized to some degree after administration with LPS-depleted OMVs or CagA-negative OMVs. The roles of ROS and NF-κB were further verified by treatment with a superoxide dismutase mimetic TEMPOL and a NF-κB inhibitor BAY11-7082. In addition, we found that *H. pylori*-derived OMVs increased the levels of inflammatory factors IL-6 and TNF-α, two downstream targets of NF-κB, in HUVECs. These suggested that ROS/NF-κB signaling pathway is involved in the *H. pylori*-derived OMVs-mediated effects in HUVECs.

It should be noted that some effects of *H. pylori* on atherosclerotic plaques are, at least in part, direct (Testerman et al., 2019). However, this does not mean that OMVs play no role in the *H. pylori*-accelerated atherosclerosis. OMVs could be transferred to the cytosol and nucleus in the recipient cells even at a distance (Keenan et al., 2008; Parker et al., 2010), indicating that OMVs generated in the stomach could be transported to the plaque, promoting atherosclerosis. It is similar with the distant effects of exosomes derived from *H. pylori*-infected gastric epithelial cells. *H. pylori* infection impairs endothelial function, induces macrophage foam cell formation and promotes atherosclerosis through exosome-mediated mechanisms (Yang et al., 2019; Xia et al., 2020). But it is also possible that OMVs are generated in the plaque and act locally although it hasn’t been reported yet. We cannot define which one could have an even greater chance of causing local damage. Moreover, OMVs were either able to cross the mucosa and enter the bloodstream or were acting at a distance, perhaps via cytokine induction. This question could be answered by determining whether CagA is present within plaques or the endothelium, which needs to be studied in the future. In addition, Testerman et al. (2019) reported that of the *H*. *pylori-*positive animals, 64% had evidence of *H*. *pylori* infection in both the stomach and arteries. Further studies showed that live *H. pylori* are frequently present in coronary arteries of *H. pylori*-infected monkeys and the density of bacteria found in plaques is strongly related to colonization density in the stomach. These indicate that the effect of OMVs-accelerated atherosclerosis may, at least in part, be associated with the amount of gastric *H. pylori* (Testerman et al., 2019).

**FIGURE 6:**
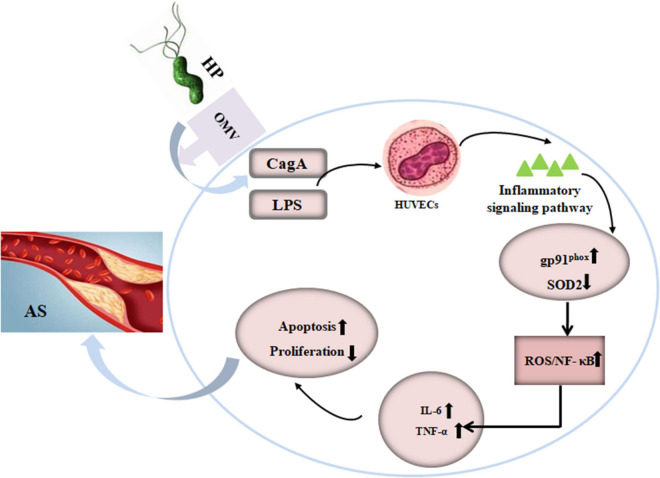
Schematic representation of the effect of OMVs from *H. pylori* on atherosclerosis plaque formation in ApoE^–/–^ mice. AS, atherosclerosis; CagA, cytotoxin-associated gene A; HP, *Helicobacter pylori*; HUVECs, human umbilical vein endothelial cells; IL-6, interleukin-6; LPS, lipopolysaccharide; NK-κB, nuclear factor-κB; OMVs, outer membrane vesicles; SOD2, superoxide dismutase 2; TNF-α, tumor necrosis factor-α.

In conclusion, we present evidence that *H. pylori*-derived OMVs accelerate atherosclerosis plaque formation via endothelium injury (Figure 6). CagA and LPS from *H. pylori*-derived OMVs, at least in part, participate in these processes. Activation of ROS/NF-κB signaling pathway may be involved in the *H. pylori*-derived OMVs-mediated effects in endothelial cells. These afford a novel mechanism between *H. pylori* infection and atherosclerosis, and suggest that preventing and eradicating the *H. pylori* infection may be a new strategy to reduce the incidence and development of atherosclerosis.

## Data Availability Statement

The original contributions presented in the study are included in the article/supplementary material, further inquiries can be directed to the corresponding author/s.

## Ethics Statement

The animal study was reviewed and approved by the Daping Hospital Animal Use and Care Committee.

## Author Contributions

NW, FZ, and CC contributed to conception and design of the study. HL organized the database. WW performed the statistical analysis. JG wrote the first draft of the manuscript. JY wrote sections of the manuscript. LL contributed to the interpretation of the data and the revision the manuscript. All authors contributed to manuscript revision, read, and approved the submitted version.

## Conflict of Interest

The authors declare that the research was conducted in the absence of any commercial or financial relationships that could be construed as a potential conflict of interest.

## Publisher’s Note

All claims expressed in this article are solely those of the authors and do not necessarily represent those of their affiliated organizations, or those of the publisher, the editors and the reviewers. Any product that may be evaluated in this article, or claim that may be made by its manufacturer, is not guaranteed or endorsed by the publisher.
